# Flow cytometry in the differential diagnosis of myelodysplastic neoplasm with low blasts and cytopenia of other causes

**DOI:** 10.3389/pore.2024.1611811

**Published:** 2024-07-08

**Authors:** Márk Plander, Mária Kányási, Tamás Szendrei, Judit Skrapits, Botond Timár

**Affiliations:** ^1^ Department of Hematology, Markusovszky University Teaching Hospital, Szombathely, Hungary; ^2^ Central Laboratory, Markusovszky University Teaching Hospital, Szombathely, Hungary; ^3^ Department of Pathology and Experimental Cancer Research, Semmelweis University, Budapest, Hungary

**Keywords:** flow cytometry, myelodysplastic neoplasms, low blasts, differential diagnosis, accuracy

## Abstract

**Background:**

Myelodysplastic neoplasms (MDS) are characterized by cytopenia, morphologic dysplasia, and genetic abnormalities. Multiparameter flow cytometry (FCM) is recommended in the diagnostic work-up of suspected MDS, but alone is not sufficient to establish the diagnosis. Our aim was to investigate the diagnostic power of FCM in a heterogeneous population of patients with cytopenia, excluding cases with increased blast count.

**Methods:**

We analyzed bone marrow samples from 179 patients with cytopenia (58 MDS, 121 non-MDS) using a standardized 8-color FCM method. We evaluated the sensitivity, specificity, and accuracy of several simple diagnostic approaches, including Ogata score, extended Ogata score, the WHO and ELN iMDSFlow recommended “3 aberrations in two cell compartments method,” and the combination of the Ogata score and “3 aberrations in two cell compartments method.” The patients were followed until the diagnosis was confirmed, with a median follow-up of 2 months (range 0.2–27).

**Results:**

The combination of Ogata score and “3 aberrations in two cell compartments method” achieved the highest diagnostic accuracy (78%) with sensitivity and specificity 61% and 86%, respectively. When using only the “3 aberrations in two cell compartments method,” the accuracy was 77% with a sensitivity of 72% and a specificity of 79%. The most frequently observed etiologies among the false positive cases were substrate deficiencies, inflammation/infection, or toxic effects. MDS can be excluded in all these cases after a thorough clinical evaluation and a relatively short follow-up.

**Conclusion:**

FCM remains an important but supplementary part in an integrated diagnostic process of MDS with low blasts. The combination of the Ogata score and the “3 aberrations in two cell compartments method” slightly improves accuracy compared to the detection of “3 aberrations in two cell compartments method” alone.

## Introduction

Myelodysplastic neoplasms (MDS) are a group of clonal haematopoietic neoplasms defined by cytopenias and morphological dysplasia [1]. Based on recurrent chromosomal abnormalities MDS can also be confirmed in a cytopenic patient, even in the absence of diagnostic morphological dysplasia [2]. The recommended threshold for dysplasia is set at 10% for all lineages [1, 2]. The MDS entities are grouped in the new World Health Organization (WHO) Classification according to genetic events and morphological aberrancies [1]. The morphologically defined categories are based on the blast count with the longstanding cut-off of 5% in the bone marrow (BM) distinguishing cases with low or high blasts. The myeloblast percentage should be determined by counting well-prepared, cellular BM aspirate smears and/or touch preparations and a peripheral blood (PB) smear [2]. To discriminate MDS cases with low blasts from reactive cytopenia is frequently a diagnostic challenge for more reasons: dysplasia over 10% of cells may occur in nonneoplastic causes of cytopenia [3, 4]; dysplasia is often subtle and not recognizable necessitating a repeated bone marrow examination [2]; identification of dysplasia is not always reproducible even among experienced hematopathologists [4–6] and cytogenetic abnormalities specific for MDS are present only in about 50% of cases [7]. Because of these difficulties other approaches such as molecular genetics and immunophenotyping can assist in the diagnosis of MDS, although neither flow cytometry nor somatic mutations alone are considered diagnostic for MDS by the WHO Classification. Recently, a prospective study identified 17 immunophenotypic aberrations that were independently associated with MDS/CMML [8]. However, it is not a single immunophenotypic aberration but a combination of different parameters that is indicative of MDS [9]. Therefore, both the WHO and the ELN iMDSFlow recommend that aberrant findings in at least three tested features affecting at least two cell compartments are highly associated with MDS [2, 10]. In addition to this recommendation, many diagnostic MDS FCM-scores such as Ogata-score [11], extended Ogata-score [12], RED-score [13], ELN-NEC [14], FCSS [15], integrated flow-score (iFS) [16] have been developed to differentiate MDS from other cytopenias. The Ogata-score with four parameters including the percentage of myeloblasts and B-progenitors, the ratio of the lymphocyte to myeloblast CD45 MFI and the granulocyte to lymphocyte SSC mode is recommended by the iMDSFlow for screening purposes [10]. Most of the FCM scores focus on only a part of hematopoiesis, except iFS, which analyzes 44 parameters including all four cell compartments [16].

Our aim was to investigate the diagnostic power of a standardized 8-color FCM method in a heterogeneous population of patients with cytopenia, excluding cases with increased blast count (myeloid progenitors≥5%).

## Materials and methods

### Patients

Bone marrow samples sent for routine immunophenotyping from cytopenic patients with suspected MDS between 01-Nov-2019 and 01-Sep-2022 were included in the study. The definition of cytopenia was at least one of the following: absolute neutrophil count <1.8 × 10^9^/L, platelet count <100 × 10^9^/L, hemoglobin concentration <100 g/L. None of the patients had received vitamin supplementation or growth factors prior to the diagnostic bone marrow aspiration, and none had received therapy for MDS during the follow-up. In all cases, bone marrow smear was evaluated in parallel with FCM, and in most cases, cytogenetics and trephine biopsy were also performed. Flow cytometry and cytomorphology were analyzed independently. Cases with myeloid progenitors ≥5% detected by either bone marrow smear or flow cytometry (FCM) and with inadequate material for cytomorphological analysis were excluded. The patients were followed until the diagnosis of MDS was confirmed. This was based on the following criteria: 1. the presence of cytogenetic aberration except for loss of chromosome Y; 2. ≥5% myeloblast in the trephine biopsy; 3. no improvement in the blood counts during the follow-up, and MDS diagnosis confirmed by a second bone marrow examination or no other etiology was found.

All patients provided informed consent for the diagnostic analysis and for the follow-up. The study was conducted in accordance with the Declaration of Helsinki and was approved by the institutional Review Board of Markusovszky University Teaching Hospital.

### Multiparameter flow cytometry

The monoclonal antibody (mAb) panel consisted of three 8-color tubes (Table 1) designed according to the iMDSFlow guidelines [10, 17]. EDTA was used as an anticoagulant in all samples. Samples were processed within 24 h after the bone marrow aspiration. For labeling, the samples were incubated with mAbs for 15 min at room temperature in the dark according to the manufacturers’ data sheets. Red blood cells were lysed using FACS lysing solution containing 1.5% formaldehyde (BD Biosciences). After washing twice in PBS, the cells were resuspended in 500 μL of PBS and measured within one hour. Samples were measured on FACSCanto II cytometer (BD Biosciences), with 50000 events acquired. Data acquisition and analysis were performed using FACSDiva software (BD Biosciences). A hierarchical gating strategy was employed for data analysis. Initially, doublets were excluded based on forward scatter area (FSC-A) versus forward scatter width (FSC-W). Subsequently, debris were excluded based on forward scatter versus side scatter (FSC vs. SSC). Finally, the main bone marrow cell lineages were gated. Monocytopoesis was identified by CD33^int^ CD64^int^, granulopoesis by CD15^+^, nucleated erythropoetic cells by CD45^dim/neg^SSC^low/int^CD33^−^CD235a^+^CD71^+^. The ratio of myeloid progenitors was enumerated by gating the CD45^dim^SSC^low/int^CD34^+^CD13^+^ and CD45^dim^SSC^low/int^CD117^ +^CD33^+^populations using the total number of nucleated cells as the denominator. In instances where the ratios differed between the two gating strategies, the higher ratio of myeloid progenitors was utilized.

**TABLE 1:** Monoclonal antibody panel.

	FITC	PE	PerCP-Cy5.5/PC5.5	PC7	APC	APC-H7	Pacific Blue	Pacific Orange
Monocyte	CD14	CD11b	CD33	CD56	CD300e	CD64	HLADR	CD45
Granulocyte	CD15	CD11b	CD34	CD13	CD10	CD16	CD7	CD45
Eryhtroid	CD71	CD117	CD33	CD105	CD36	CD235a	HLADR	CD45

### Diagnostic MDS FCM-scores and thresholds

The Ogata score [11] and extended Ogata score [12] were counted and the thresholds of MDS were set to ≥2 for both scores. For Ogata score, four parameters were analyzed (1 point each): the percentage of myeloblasts in all nucleated cells (cut-off ≥2%), the percentage of B-progenitors in all CD34^+^ cells (cut-off ≤5%), the lymphocyte to myeloblast CD45 MFI ratio (≤4 or ≥7.5), and the granulocyte to lymphocyte SSC mode ratio (cut-off ≤6). In the case of extended Ogata score, one extra point was added for the expression of CD7 on myeloid progenitors with a threshold of 20% or of CD56 on monocytes, with a threshold of 30% positive cells.

In addition to these scores, the presence of at least three aberrations in at least two cell compartments was considered indicative of MDS. During the data analysis predefined templates and gates were applied in the FACSDiva software to assess the distinction from the normal pattern. The sensitivity, specificity, and accuracy of Ogata score, extended Ogata score, “3 aberrations in two cell compartments method” and the combination of the Ogata score and “3 aberrations in two cell compartments method” were evaluated.

## Results

### Description of the study population

A total of 179 patients with cytopenia were included in the study. The median age was 72 years (range 28–90). Of these patients, 95 were female. A total of 58 cases were diagnosed as MDS. In the majority of MDS cases the mean corpuscular volume (MCV) was increased or normal in 28 and 27 cases, respectively. In 15 cases, cytogenetic aberrancies in addition to morphological dysplasia were observed. The detected chromosomal abnormalities were complex in 7 cases, del (5q) alone in 4 cases, del (20q) in 2 cases, trisomy 8 (+8) in 1 case, del (9q) in 1 case. In five cases, a ≥5% myeloblast count was found in the trephine biopsy. In 38 cases with morphological dysplasia, there was no improvement in the blood counts during the follow-up period and the diagnosis was confirmed by a second bone marrow examination and/or by excluding other etiologies. Of these 38 patients, ring sideroblasts in 7 cases, increasing ratio of myeloblasts in 3 cases or progression of cytopenia during follow-up in 4 cases, clonal chromosomal (not MDS defining) abnormality in 2 cases, abnormal monocytosis and granulocytosis in 1-1 cases, and dysplasia in more than one lineage in 12 cases were indicative of MDS. The median follow-up period was 2 months (range 0.2–27). MDS was classified according to the 2016 WHO classification: MDS with isolated del (5q) 4 (7%), MDS with single lineage dysplasia (SLD) 5 (9%), MDS with multilineage dysplasia (MLD) 34 (57%), MDS with ring sideroblasts and single lineage dysplasia (RS-SLD) 3 (5%), MDS with ring sideroblasts and with multilineage dysplasia (RS-MLD) 5 (9%), MDS with excess blasts (EB1) 5 (9%), chronic myelomonocytic leukaemia (CMML) 1, myelodysplastic/myeloproliferative neoplasm, unclassifiable (MDS/MPN-U) 1.

The etiology of non-MDS cytopenias is listed in Table 2. The most common causes are vitamin B12 deficiency, toxic effects of drugs, inflammation, and chronic myeloproliferative neoplasia, with primary myelofibrosis being the most prevalent. In many cases, a combination of different causes was diagnosed. In non-MDS cases, a normal MCV was observed most frequently then increased and decreased in 59, 52, and 10 cases, respectively.

**TABLE 2:** Etiology of non-MDS cytopenia.

Etiology	Number of cases (n = 121)
Chronic kidney disease	9
Inflammatory	12
Vitamin B12 deficiency	16
Toxic (drugs, alcohol)	16
ITP	6
Aplastic anaemia	6
Iron deficiency, bleeding	6
Myeloproliferativ neoplasms	12
Autoimmune (SLE, PRCA, CIN)	7
Liver cirrhosis, hypersplenism	3
Solid tumor, T-LGL	3
Combined	19
Unknown	6

### Myeloid progenitors

**FIGURE 1:**
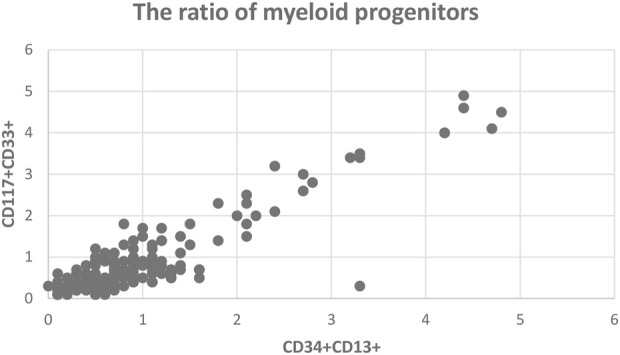
The ratio of myeloid progenitors by two different gating strategy.

The ratio of myeloid progenitor cells (MPCs) was determined by gating the CD45^dim^SSC^low/int^CD34^+^CD13^+^ or CD45^dim^SSC^low/int^CD117^+^CD33^+^ populations, which correlated in most of the cases well (Figure 1). In MDS cases, the mean ratio of MPCs with the two different gating strategies was 1.6% (range 0%–4.7%) and 1.6% (range 0.1–4.6%), respectively. A difference in the MPCs exceeding 1% between the two gating strategies was observed in only 2 cases (3.4%) due to loss of either CD34 or CD33.

In non-MDS cases, the ratio of MPCs by the two different gating approaches was 0.8% (range 0.1–2.7%) and 0.7% (range 0.1%–3%), respectively. Myeloid progenitors over 2% were detected in five patients: 2 cases with pancytopenia due to methotrexate, 2 cases with myeloproliferative neoplasia (MPN), and one case with infection.

### Sensitivity of FCM approaches

The sensitivity, specificity, and accuracy of the Ogata score, the extended Ogata score, the “3 aberrations in two cell compartments method” and the combination of the Ogata score with the “3 aberrations in two cell compartments method” were calculated.

The Ogata score was not applicable in only one MDS case due to the loss of CD34 expression on myeloid progenitors. The sensitivity of the Ogata score proved to be 75%. Regarding the 14 false negative cases, 12 of them belonged to the low or very low risk group by R-IPSS, with normal karyotype and myeloid progenitors ≤2% by either method (BM smear, trephine biopsy or FCM). One of the remaining two cases was MDS with fibrosis and complex cytogenetics, while the other was an MDS/MPN with 4% myeloblast in the trephine biopsy. The sensitivity of the extended Ogata score was improved to 81% by identifying an additional 3 MDS cases.

The “3 aberrations in two cell compartments method” yielded a sensitivity of 72%. Sixteen cases were identified as false negatives. Eight of these cases were classified as low or very low risk according to the R-IPSS, while three were placed in the intermediate risk group. In five cases, the R-IPSS could not be calculated due to unsuccessful karyotyping. Two of these cases were classified as MDS-EB1 based on the increased blasts observed in the trephine biopsy.

The sensitivity decreased to 61% when the Ogata score and the “3 aberrations in two cell compartments method” were combined, classifying the cases as MDS if both turned out to be suggestive of MDS (Table 3).

**TABLE 3:** The sensitivity for different FCM approaches in MDS. Ogata score and extended Ogata score are not applicable in one case.

	MDS Sensitivity	
	Number of patients (n = 58)	Percentage of patients (%)
Ogata-score	43	75
Extended Ogata-score	46	81
ELN	42	72
Ogata-score + ELN	35	61

### Specificity and accuracy of FCM approaches

In patients with non-MDS cytopenia all four FCM approaches showed a specificity of at least 62%. The Ogata score and the extended Ogata score exhibited the same specificity. Regarding the Ogata score, score 2 was observed with high frequency in 36 false positive cases. Scores 3 and 4 were more specific and were found in only 10 of non-MDS cases. The decreased B-progenitor cluster size and reduced granulocyte/lymphocyte SSC ratio were detected in 43 (93%) and 33 (72%) of the false positive non-MDS cases, respectively. These two aberrations were observed in cases of any etiology. The decreased B-progenitor cluster size occurred most often in elderly (>70 years), in autoimmune cytopenia and in inflammatory diseases, while reduced granulocyte/lymphocyte SSC ratio was most often found in cytopenia due to toxic effects and inflammation. Abnormal lymphocyte to myeloblast CD45 ratio and increased myeloblast ratio were shown in 15 (32%) and 4 (9%) false positive non-MDS cases, respectively. The most common etiology for the high (3 and 4 points) Ogata scores was pancytopenia due to toxic effects from methotrexate (4 cases). The specificity of the “3 aberrations in two cell compartments method” was 79%. The majority of the false positive cases were attributed to substrate deficiencies, inflammation/infection, and toxic effects. The combination of the Ogata score with the “3 aberrations in two cell compartments method” allowed the exclusion of the majority of non-MDS cases with a specificity of 86% (Table 4). The reasons for false positivity with the combination approach were variable. These included vitamin B12 deficiency (4 cases), inflammation/infection (3 cases), toxic effects (3 cases), combination of different causes (4 cases) (Table 5).

**TABLE 4:** The specificity for different FCM approaches in non-MDS cases.

	Non-MDS Specificity	
	Number of patients (n = 121)	Percentage of patients (%)
Ogata-score	75	62
Extended Ogata-score	75	62
ELN	96	79
Ogata-score + ELN	104	86

**TABLE 5:** Diseases resulted in false positivity with the different FCM approaches.

Etiology	Ogata score	ELN	Ogata score + ELN
Substrate deficiency	9	8	4
Inflammatory	9	4	3
Toxic	6	3	3
Autoimmune	6	3	1
Myeloproliferative neoplasms	3	2	1
T-LGL	1	1	1
Chronic kidney disease	2	0	0
Chronic liver disease	2	0	0
Combined	8	4	4

Interestingly, in 12 cases, dysplastic changes in the bone marrow smear were observed without an increase in blasts or cytogenetic aberrancies. These cases demonstrated improvement in blood counts during the follow-up period, which led to the exclusion of MDS. In half of these cases, FCM demonstrated the absence of signs of MDS using any method, while in only three cases, MDS was identified based on the combination of the Ogata score and the “3 aberrations in two cell compartments” method.

The highest accuracy (78%) was achieved by the combination of the Ogata score and the “3 aberrations in two cell compartments method.” The application of the “3 aberrations in two cell compartments method” resulted in almost the same accuracy (77%) (Table 6).

**TABLE 6:** The accuracy of different FCM approaches in patients with cytopenia. Ogata score and extended Ogata score are not applicable in one case.

	MDS and non-MDS Accuracy	
	Number of patients (n = 179)	Percentage of patients (%)
Ogata-score	118	66
Extended Ogata-score	121	68
ELN	138	77
Ogata-score + ELN	139	78

### Results of the repeated bone marrow examination

In 9 cases the bone marrow examination was repeated after a median of 11 months (range 7–24). In 3 cases, an increasing ratio of myeloblasts was detected in the second bone marrow sample (2 MDS-EB2 and 1 AML). In 2 cases, both the Ogata and the ELN scores were already abnormal in the first bone marrow sample and the myeloblast ratio did not increase in the second sample. In 3 cases, the Ogata score and in one case the ELN score became abnormal in the second sample. As a result of these changes, both scores became abnormal in two patients.

## Discussion

The differential diagnosis of myelodysplastic neoplasms with low blasts represents a diagnostic challenge in patients presenting with cytopenia. The gold standard in the diagnosis is the evaluation of bone marrow smears for dysplastic features. Even in the absence of morphological dysplasia, several chromosomal abnormalities and two mutations—biallelic TP53 and SF3B1 mutations—are considered MDS defining as per the WHO 5th edition (WHO5ED) criteria [1]. An abnormal immunophenotype alone is only a co-criterion for the diagnosis of MDS [18]. FCM, however, is widely available and provides results in a relatively short time frame, typically within 24 h. Despite the efforts of iMDSFlow in the standardization of flow cytometry in MDS there are difficulties in the application due to the large number of proposed markers (20 antigens). Furthermore, none of the markers are specific to MDS, and there are no clear cut-off values for most of these markers [10, 17]. A number of FCM scoring systems have been published and validated for the analysis of MDS [11–16, 19]. The goal of these scoring systems was to determine the most relevant markers for the evaluation and to establish cut-offs that best discriminate between MDS and non-MDS cases. Recently, two studies compared the most important scoring systems and not surprisingly, the most comprehensive one, iFS, proved to be the most useful [20, 21]. iFS combines a modified FCSS, Ogata-score and ELN-NEC requiring the analysis of 44 markers and calculation of 3 scores [16]. Therefore, the application of iFS is labor-intensive and time-consuming. A survey evaluating the current FCM practice for the diagnosis of MDS in 229 laboratories worldwide showed that the usage of any FCM scoring system was very limited [22]. This result reflects that calculating the scores is hardly compatible with the routine clinical practice. In the present work, we tested the simplest FCM approaches for the diagnosis of MDS such as the Ogata score, the extended Ogata score, and the ELN and the WHO recommended “3 aberrations in two cell compartments method.” An important feature of the Ogata score is its lower sensitivity in low-risk MDS patients. Therefore, we investigated whether the combination of the Ogata score with the “3 aberrations in two cell compartments method” improves this sensitivity.

Furthermore, we focused on the most problematic group of MDS (MDS with low blasts), excluding cases with myeloid progenitors≥5% detected by either bone marrow smear analysis or FCM. Follow-up of these patients, even with morphologic dysplasia allowed confirmation of the diagnosis in all cases. The follow-up seemed necessary because in 12 cases the bone marrow smear showed dysplastic changes, but the blood counts normalized suggesting reactive causes in the background. This finding confirms that dysplastic changes are not specific for MDS [3, 4] and the need for follow-up.

The identification of myeloid progenitor cells (MPCs) represents a pivotal aspect in the diagnosis and prognostication of MDS. An aberrantly low expression of CD34 on MPCs may occur in MDS [23], therefore two distinct gating strategies for MPCs were employed: one based on CD34 expression and the other on CD117 and CD33 expression. Using this approach, we were able to identify MPCs even in cases with loss of CD34 or CD33, otherwise the results correlated perfectly. In cytomorphology, a cut-off of 5% blasts is used for diagnosis and classification. However, there are striking differences in overall survival and AML evolution depending on blast counts even under 5% [24]. A lower cut-off of myeloid progenitors is recommended for FCM in MDS diagnosis. In the Ogata-score, a 2% cut-off is applied for myeloblast-related cluster size in all nucleated cells [11]. In a prospective FCM study, MPCs over 3% among the CD45^+^ cells were associated with the diagnosis of MDS [8]. We confirmed these findings because in all non-MDS cases the ratio of MPCs was at a maximum of 3%, although it should be noted that the results are not completely comparable due to the use of all nucleated cells as a denominator. MPCs above 2% were found in some patients with regenerating myelopoiesis after drug-induced pancytopenia or in MPNs.

One of the diagnostic tools we selected was the Ogata score, which was chosen for its simplicity. This score eliminates errors deriving from the inter-observer variability. The sensitivity of the Ogata score was found to be slightly higher (75%) in our hands than in the previous studies [11, 12, 21]. The sensitivity of the Ogata score is the lowest in low risk cases [12, 21], as evidenced by the fact that almost all false negative cases in our patient group were classified as IPSS-R low or very low risk. CD5 was excluded from the extended Ogata score due to its low sensitivity. The addition of CD56 and CD7 allowed the identification of 3 more MDS patients, thereby improving the sensitivity of the Ogata score in a manner similar to that reported in the original publication [12].

The sensitivity of the “3 aberrations in two cell compartments method” was somewhat lower than that of the Ogata-score, missing 16 cases, including 5 with either intermediate risk category or higher blast counts. These higher risk cases were correctly diagnosed with the Ogata-score, suggesting that these two methods are to some extent complimentary. When both a positive Ogata-score and a “3 aberrations in two cell compartments method” were required for MDS diagnosis, the sensitivity decreased to 61%.

The most surprising finding of our analysis is the low specificity of the Ogata score. In previously published studies, this score demonstrated at least 80% specificity making it an effective tool for excluding the majority of non-MDS cases [11, 12, 19–21]. However, in our analysis, even score 4 was not entirely specific for MDS, in contrast to the findings of previous studies [11, 25]. The most common parameter in non-MDS cases was the decreased B-progenitor cluster size, as previously described in the literature [11, 25]. The older age of our patient population [26], the high frequency of inflammation [27], autoimmunity and its treatment by drugs such as metothrexate [28] may explain the high prevalence of low numbers in B-progenitors. The reduced granulocyte/lymphocyte SSC ratio was the second most common parameter in our non-MDS cohort, although this is considered to be MDS specific and is associated with the defective maturation of myeloid precursors [29]. Our methodology may contribute to the high rate of reduced granulocyte/lymphocyte SSC ratio, because FACS Lysing solution (BD) was used to lyse the non-nucleated red blood cells. ELN iMDSFlow recommends ammonium chloride [17], and it was also used in the original publication of the Ogata score [30]. FACS Lysing Solution and a stain-lyse-wash protocol, which we used, significantly reduce the light scatter CV for the different leukocyte populations compared to ammonium chloride [31], and may therefore influence the granulocyte/lymphocyte SSC ratio. However, this staining procedure did not decrease the sensitivity of the Ogata score in our cohort, and Matzen et al also used FACS Lysing solution with high specificity in their MDS analysis [32], so the FACS Lysing solution is unlikely to have a significant effect on the SSC ratio. The specificity of the “3 aberrations in two cell compartments method” was found to be higher (79%), although this figure is still far from 100%. This result confirms that the altered patterns of antigen expression observed in MDS are not specific for this disease and are susceptible to misinterpretation.

The combination of the Ogata score and the “3 aberrations in two cell compartments method” yielded the highest accuracy, correctly identifying 79% of the cases. The application of the “3 aberrations in two cell compartments method” resulted in an almost identical accuracy.

In conclusion, this study confirms that the diagnosis of MDS with low blasts should be an integrated approach that includes morphology, cytogenetics, immunophenotyping and molecular genetics. Flow cytometry can support the diagnosis of MDS, but it does not provide a definitive diagnosis or exclude the possibility of MDS. This work emphasizes the importance of patient follow-up, as even dysplastic morphology and indicative FCM results can be misleading.

## Data Availability

The datasets presented in this article are not readily available because the dataset is anonymized and not available for the public. Requests to access the datasets should be directed to planderm@yahoo.com.
