# Supplementary material for: Flow cytometry in the differential diagnosis of myelodysplastic neoplasm with low blasts and cytopenia of other causes
Source: Pathol Oncol Res. 2024 Jul 8;30:1611811. doi: 10.3389/pore.2024.1611811 (PMC11260641; doi:10.3389/pore.2024.1611811)
Supplement: Supplementary file 1 [file Table1.docx]

Supplementary table 1.

Overview of the analyzed parameters

| Bone marrow subset | Analysis | Aberrancy |
| --- | --- | --- |
| Immature myeloid and monocytic progenitors | Percentage of cells in nucleated cell fraction | Increased |
|  | Expression of CD45 | Lack of/decreased/increased |
|  | Expression of CD34 | Lack of/decreased/increased |
|  | Expression of CD117 | Homogenous under/overexpression |
|  | Expression of HLA-DR | Lack of/increased |
|  | Expression of CD13 and CD33 | Lack of/decreased/increased |
|  | Asynchronous expression of CD11b, CD15 | Presence of mature markers |
|  | Expression of CD7, CD56 | Presence of lineage infidelity markers |
| Maturing neutrophils | Percentage of cells as ratio to lymphocytes | Decreased |
|  | SSC as ratio vs SSC of lymphocytes | Decreased |
|  | Relationship of CD13 and CD11b | Altered pattern |
|  | Relationship of CD13 and CD16 | Altered pattern |
|  | Relationship of CD15 and CD10 | Altered pattern |
| Monocytes | Distribution of maturation stages | Shift towards immature |
|  | Relationship of HLA-DR and CD11b | Altered pattern |
|  | Expression of CD13 and CD33 | Homogenous under/overexpression |
|  | Expression of CD56 | Presence of lineage infidelity markers |
| Erythroid compartment | Relationship of CD71 and CD235 | Altered pattern |
|  | Expression of CD71 | Decreased |
|  | Expression of CD71 | Increased CV |
|  | Expression of CD36 | Decreased |
|  | Expression of CD36 | Increased CV |
|  | Percentage of CD117-positive precursors | Decreased/increased |
|  | Expression of CD105 | Altered pattern |
